# Characteristics and Outcomes of Liver Transplantation Recipients after Tranexamic Acid Treatment and Platelet Transfusion: A Retrospective Single-Centre Experience

**DOI:** 10.3390/medicina59020219

**Published:** 2023-01-23

**Authors:** Mohmad H. Alsabani, Abdulrazak Sibai, Saja F. Alharbi, Lafi H. Olayan, Abeer A. Samman, Mohammed K. Al Harbi

**Affiliations:** 1 Anesthesia Technology Department, College of Applied Medical Sciences, King Saud bin Abdulaziz University for Health Sciences, Riyadh 11481, Saudi Arabia; 2 King Abdullah International Medical Research Centre, Riyadh 11481, Saudi Arabia; 3 Department of Anesthesia, Ministry of National Guard Health Affairs, Riyadh 11426, Saudi Arabia; 4 College of Medicine, King Saud bin Abdulaziz University for Health Sciences, Riyadh 11481, Saudi Arabia

**Keywords:** tranexamic acid, platelet transfusion, liver transplantation, blood product transfusion, hepatic artery thrombosis, portal vein thrombosis, postoperative complications

## Abstract

*Background and Objectives*: Patients undergoing liver transplantation (LT) often require increased blood product transfusion due to pre-existing coagulopathy and intraoperative fibrinolysis. Strategies to minimise intraoperative bleeding and subsequent blood product requirements include platelet transfusion and tranexamic acid (TXA). Prophylactic TXA administration has been shown to reduce bleeding and blood product requirements intraoperatively. However, its clinical use is still debated. The aim of this study was to report on a single-centre practice and analyse clinical characteristics and outcomes of LT recipients according to intraoperative treatment of TXA or platelet transfusion. *Materials and Methods*: This was a retrospective observational cohort study in which we reviewed 162 patients’ records. Characteristics, intraoperative requirement of blood products, postoperative development of thrombosis and outcomes were compared between patients without or with intraoperative TXA treatment and without or with platelet transfusion. *Results*: Intraoperative treatment of TXA and platelets was 53% and 57.40%, respectively. Patients who required intraoperative administration of TXA or platelet transfusion also required more transfusion of blood products. Neither TXA nor platelet transfusion were associated with increased postoperative development of hepatic artery and portal vein thrombosis, 90-day mortality or graft loss. There was a significant increase in the median length of intensive care unit (ICU) stay in those who received platelet transfusion only (2.00 vs. 3.00 days; *p* = 0.021). Time to extubate was significantly different in both those who required TXA and platelet transfusion intraoperatively. *Conclusions*: Our analysis indicates that LT recipients still required copious intraoperative transfusion of blood products, despite the use of intraoperative TXA and platelets. Our findings have important implications for current transfusion practice in LT recipients and may guide clinicians to act upon these findings, which will support global efforts to encourage a wider use of TXA to reduce transfusion requirements, including platelets.

## 1. Introduction

Since the first liver transplantation (LT) which was carried out in the 1960s, numerous LT surgeries have been performed throughout the past decades. Nowadays, with enhancements in anaesthesia care and surgical techniques, LT has shifted from a high-risk operation to a routine surgical procedure. However, a major remaining challenge for the transplantation community is the massive blood loss during the surgery [1,2,3,4]. To overcome this challenge, transfusion of blood components and tranexamic acid (TXA) treatment have been used to compensate for blood loss through volume replacement and bleeding minimisation, respectively [5]. TXA, an antifibrinolytic agent, has been widely investigated in various surgical settings including orthopaedics [6,7,8], obstetrics [9,10,11], cardiology [12,13], and LT [14,15,16]. Nevertheless, its clinical use is still limited in current practice due to serious complications concerning thrombosis formation. Furthermore, Walsh and colleagues have reported geographical variations in the use of TXA among trauma patients [17]. Countries of Asia and Africa were among those who have reported a lower use of TXA.

Despite the clinical benefits of blood product transfusion in managing bleeding patients, allogenic blood product transfusion has substantial side effects on postoperative outcomes [4,18,19,20,21]. Platelet transfusion has been identified as a factor with detrimental effects on patients’ outcomes [3,22,23,24,25]. Intraoperative transfusion of 20 or more units of platelets demonstrated a marked increase in both intensive care unit (ICU) and hospital length of stay [26]. More recently, platelet transfusion was associated with early and 90-day mortality in living donor liver transplant recipients [24]. Furthermore, platelet transfusion has been identified as a significant prognostic factor for patient survival as well as graft loss [3].

The timing of TXA administration has been reported previously as a critical factor that influences the efficacy of this drug [27,28]. Previous prospective studies have established the role of prophylactic TXA during LT to reduce bleeding and the subsequent transfusion requirements of blood products, including platelets [14,15]. However, data on the efficacy of intraoperative administration of TXA and/or platelets in LT are still limited. More recently, scientists and clinicians are taking initiatives towards a wider implementation of TXA in surgical settings [29,30]. Hence, this retrospective study aimed to identify whether intraoperative administration of TXA and/or platelets influences intraoperative blood transfusion requirements, the development of postoperative thrombotic events and overall outcomes of LT recipients. We hypothesised that patients treated with TXA and/or platelets intraoperatively would still require substantial amounts of blood products during surgery.

## 2. Materials and Methods

### 2.1. Design and Participants

This is a retrospective, observational cohort study in a single centre of patients who underwent LT from 2016 to 2020. We included all adult patients who underwent LT surgery for the first time during the period between 2016–2020. Exclusion criteria included patients with coagulopathies, paediatric transplant (age < 18) and patients who underwent re-transplantation or combined organ transplantation.

### 2.2. Data Collection and Grouping

The data were retrieved from electronic health records of the patients via the hospital’s data system. The study’s primary outcome was intraoperative transfusion requirements of blood products. The secondary outcomes were ICU length of stay, postoperative time to extubate, incidence of postoperative hepatic artery thrombosis (HAT) and portal vein thrombosis (PVT) and the survival at 90 days.

We collected baseline data of patients which were age, gender, body mass index (BMI), model for end-stage liver disease (MELD) score, haemoglobin level, platelets, neutrophil and lymphocyte counts and international normalised ratio (INR). We also collected intraoperative variables including total intraoperative fluid replacement with crystalloids and/or colloids, requirement of TXA treatment with total dose and total blood product transfusion requirement of packed red blood cells (PRBC), fresh frozen plasma (FFP), platelet, cryoprecipitate, cell saver red blood cells (RBC) and fibrinogen concentrate. Postoperative variables were ICU length of stay, time until extubation, postoperative development of HAT and PVT and the survival at 90 days. We grouped the patients according to intraoperative treatment of TXA, platelet transfusion or combined treatment of TXA and platelets.

### 2.3. Definitions

Patient survival is defined as the time period between transplantation and the end of follow-up or patient death. HAT (ICD-10 code I74.8) is defined as clot formation in the hepatic artery that causes a blockage or narrowing of the artery, while PVT refers to the complete or partial obstruction of blood flow in the portal vein due to the presence of a clot. The incidences of HAT and PVT were documented in patients’ examinations using Doppler and computed tomography (CT) scan records.

### 2.4. Statistical Analysis

Distributions of continuous variables were assessed using the Shapiro–Wilk test. Continuous variables were non-normally distributed and are presented as the medians and interquartile ranges (IQR). Categorical variables were reported as numbers of cases and percentages. Pearson’s chi-square test or Fisher’s exact test were used to compare categorical variables as appropriate, while continuous variables were compared using the Mann–Whitney U-test. A *p*-value less than 0.05 (2-sided) was considered as statistically significant. All statistical analysis was performed using the SPSS 28.0v (IBM SPSS, Inc., Chicago, IL, USA) program.

## 3. Results

### 3.1. Perioperative Characteristics and Outcomes of Patients Categorised by Intraoperative Requirement of TXA

A total of 162 patients who underwent LT recipient surgery were enrolled in this study. Patients were grouped according to intraoperative administration of TXA. Of these patients, 86 (53.09%) received intraoperative TXA treatment. Demographics and clinical characteristics of the study groups are shown in Table 1. No significant differences were found between groups regarding gender, BMI, MELD score, haemoglobin, platelets, lymphocytes or INR. Most patients in both groups received similar intraoperative fluid management of crystalloids and colloids. Median age was slightly increased, and median neutrophils count was decreased in the TXA group (59 vs. 58 years, *p* = 0.024, and 2.30 vs. 3.89, *p* < 0.001, respectively).

To investigate intraoperative transfusion requirements after TXA administration, we compared total blood product requirements in LT patients who did not receive or received TXA treatment. As shown in Table 2, intraoperative blood product requirements of nearly all blood components were significantly higher in patients treated with TXA. Compared with patients who did not receive intraoperative TXA (non-TXA group), the TXA group had a significant increase in the requirements of PRBCs (*p* = 0.001), platelets (*p* <0.001), cryoprecipitates (*p* = 0.004), FFP (*p* <0.001) and cell saver RBCs (*p* = 0.013). However, there was no significant difference in fibrinogen concentrate requirements between both groups (*p* = 0.063).

Findings regarding post-transplant complications and outcomes are reported in Table 3. In comparison with patients who received no TXA, we observed no significant differences in the postoperative development of HAT, PVT, graft loss, length of ICU stay or 90-day mortality. Among 86 patients who received TXA treatment, only 2 patients (2.30%) developed HAT and PVT. Furthermore, 14 patients (16.30%) had graft loss postoperatively. The overall mortality rate was slightly higher (8.10%) in the TXA group compared to the non-TXA group (2.60%). Although we observed a statistically significant association in time to extubate, this observation might not be clinically meaningful.

### 3.2. Demographics, Clinical Characteristics and Outcomes of Patients Requiring Intraoperative Platelet Transfusion

Of the 162 patients, 93 received intraoperative platelet transfusion during LT surgery. As shown in Table 4, there were no significant differences in age, gender or BMI. Compared with those who did not receive intraoperative platelet transfusion, the platelets group had a higher median MELD score (21 vs. 20; *p* = 0.007). Preoperative laboratory variables were significantly different between both groups. More than half of the patients who received intraoperative platelet transfusion required TXA treatment compared to 37.68% in the other group (*p* < 0.001). The median dose of TXA treatment was also significantly higher in the platelets group compared to the non-platelets group (*p* < 0.001). Among those who received platelets intraoperatively, median transfusion requirements of all blood products were significantly higher.

Table 5 lists postoperative complications and the outcomes of patients who did not receive and those who did receive intraoperative transfusion of platelets. No significant difference was found between the two groups with respect to postoperative development of HAT and PVT (4.34% vs. 6.45%, *p* = 0.733, and 2.90% vs. 3.22%, *p* = 1.00, respectively). However, there were significant differences in time to extubate and postoperative length of ICU stay. Patients who required intraoperative platelet transfusion had an increased length of ICU stay compared to those who did not receive platelets (median: 3.00 vs. 2.00 days; *p* = 0.021). Furthermore, we observed a significant association between time to extubation and intraoperative platelet transfusion (*p* = 0.002). Graft loss was approximately similar between both groups (12.90% vs. 11.59%; *p* = 0.815). The overall 90-day mortality was increased in the platelets group compared to the no platelets group but was statistically insignificant (7.53% vs. 2.90%; *p* = 0.302).

### 3.3. Demographics, Clinical Characteristics and Outcomes of Patients Requiring Combined Intraoperative Treatment of TXA and Platelets

Of the 162 patients, 60 patients received combined intraoperative treatment of TXA and platelets. As shown in Table 6, there were no significant differences with respect to age, gender, BMI or lymphocyte count. Baseline laboratory variables were significantly different between both groups. Among those who received combined TXA and platelets intraoperatively, the median transfusion requirements of crystalloid fluid and all blood products were significantly higher.

Findings on post-transplant complications and the outcomes of patients who received both TXA and platelet treatment compared to those who did not receive either therapy are reported in Table 7. We observed no significant difference in the postoperative development of HAT, PVT, graft loss or 90-day mortality. Among 60 patients who received both treatments, we observed non-clinically relevant but statistically significant differences in time to extubate and length of ICU stay.

## 4. Discussion

In our retrospective analysis, we investigated the intraoperative requirements of blood products and the postoperative development of thrombotic events and overall outcomes according to intraoperative TXA treatment, platelet transfusion or combined treatment of both therapies in LT patients. Our main findings demonstrate that patients who received TXA treatment required more blood product transfusion. We did not observe any significant association that links TXA use to the development of postoperative thrombotic events or improved overall outcomes. Using the same cohort, we divided patients according to the intraoperative requirements of platelets. We also did not find any statistical differences between the postoperative development of thrombotic events and intraoperative platelet transfusion. Likewise, we did not observe any statistically significant association that links platelet use to graft loss or 90-day mortality. However, postoperative ICU stay was significantly increased in patients who received platelets.

Massive bleeding due to hyperfibrinolysis is common among patients undergoing LT surgeries, especially during the graft reperfusion phase [31]. TXA has been known to improve the outcomes of surgical patients by reducing blood loss via the inhibition of the proteolysis of fibrin blood clots by plasmin, which in turn reduces subsequent blood transfusion requirements [29,32,33,34]. The incorporation of TXA into the care of surgical patients is a quality standard by the National Institute for Health and Care Excellence (NICE) to reduce blood loss and subsequent adverse events when estimated blood loss is more than 500 mL in adults [35]. However, a lack of large-scale studies in LT surgeries and uncertainty about TXA-associated adverse events such as the development of thrombotic events have limited its surgical use. Previous studies have shown that TXA reduces blood loss by ~25% and blood transfusion requirements without increasing the risk of thrombotic events [30]. In LT, prophylactic usage of TXA can markedly decrease FFP and cryoprecipitate and platelet requirements intraoperatively [14,15,16]. Our findings demonstrated that more than half of our cohort received TXA, and those patients still required a very significant amount of PRBCs, FFP, cryoprecipitates and platelets during LT surgery. This is consistent with a previous randomised controlled trial of 32 patients, which showed that a small-dose infusion of TXA after the induction of anaesthesia did not minimise transfusion requirements in patients undergoing LT surgery. Possible explanations for our findings could be that the non-TXA group did not bleed significantly and/or the current practice in our institution is that TXA is used to manage active intraoperative bleeding but is not used as a preventative measure of anticipated blood loss. In support of this, recent evidence from the Clinical Randomisation of an Antifibrinolytic in Significant Haemorrhage (CRASH-2) trial on adult trauma patients with significant haemorrhages did not find a substantial reduction in transfusion requirements after TXA treatment [36]. In later exploratory analyses by the same research group, the authors highlighted that early administration of TXA should be considered soon after injury in trauma patients with substantial bleeding, as no significant benefit was found after delayed use [27]. Together, these observations highlight that the timing of TXA administration is a critical contributing factor that influences the effects of TXA.

Despite the antifibrinolytic properties of TXA, thromboembolic complications after TXA administration are rare [29]. A recent multi-centre trial of 9535 non-cardiac surgical patients has evaluated the safety outcomes of TXA compared with a matched placebo for the risk of composite of myocardial injury, non-haemorrhagic stroke, peripheral arterial thrombosis and symptomatic PVT. This outcome composite occurred in 14.2% of patients receiving TXA compared to 13.9% in the placebo group [30]. In line with previous studies [14,15,30], our data suggest that the administration of TXA did not show any association of TXA with immediate postoperative thromboembolic events.

Platelets are critically important for normal homeostasis. Abnormalities in platelet count and function may increase the risk of blood loss and necessitate platelet transfusion [37]. Although the role of platelets in homeostasis is well established, accumulating evidence has demonstrated that platelets’ function extends beyond their well-known role and they may be included in deleterious processes [37,38,39,40]. Furthermore, the negative impact of platelet transfusion on patient outcomes has been reported in various surgical settings, including LT [3,23,25]. In the present study, we stratified the same cohort according to the intraoperative transfusion of platelets and analysed their characteristics and outcomes. Our findings demonstrated that patients who received intraoperative platelet transfusion were sicker as shown by the MELD score, had worse preoperative laboratory values, required more intraoperative blood product transfusion and had an increased length of ICU stay, although the latter might not be clinically relevant. Our findings indicate that these patients who required platelet transfusion were sicker and had disturbed haemostatic function. These observations are similarly reported by Pereboom and colleagues who found that patients from the platelet transfusion group had a higher MELD score, and lower preoperative laboratory values of platelets, haemoglobin and prothrombin time [23].

In this study, we observed that LT recipients who required platelet transfusion also required increased transfusion of PRBCs, cryoprecipitates, FFP, cell saver RBCs and fibrinogen concentrates. We also noted that more than 60% of patients in the platelets group required TXA administration compared to ~35% in the no platelets group. Upon further analysis, we observed that patients who required combined treatment of TXA and platelets also required increased transfusion amounts of blood products. These findings confirm our previous observations which implied that patients from the platelets group had disturbed haemostatic function evidenced by disturbed preoperative laboratory values and increased fibrinolysis, evidenced by increased TXA use, and therefore required more transfusion of blood components to overcome these disturbances. The transfusion of blood products and plasma-rich blood products in specific components (e.g., platelets) can be deleterious and induce adverse complications such as transfusion-related acute lung injury (TRALI) [23]. Our findings are in line with Pereboom et al. who reported increased blood product requirements in those who received platelet transfusion [23]. However, they did not observe any differences in the use of antifibrinolytic agent, namely Aprotinin, between the platelets and the no platelets groups. Pereboom and colleagues acknowledged that they adapted guidelines concerning the efficacy of Aprotinin during their study period. Therefore, Aprotinin was administered in all subjects except for those whereby Aprotinin was contraindicated with them.

In our study, there was no difference between the platelets and the no platelets group in the development of immediate postoperative thromboembolic events, graft loss or 90-day mortality.

The current study has several limitations. First, this was a single-centre study and data were collected retrospectively. Second, the timing of TXA administration is not standardised in our institution and is based on the clinical judgement of the anaesthetist and surgeon. However, it was administered intraoperatively in all subjects. Third, the baseline platelet count is a confounding factor, and therefore may have influenced our results. Lastly, our follow-up for the development of postoperative thrombotic events was only limited to the duration of ICU and hospital stay.

Within the confines of these limitations, we believe our findings are meaningful and clinically relevant as they expand the knowledge regarding the clinical implications of intraoperative administration of TXA and platelets. It has become common practice in our centre to administer TXA intraoperatively to manage bleeding patients during LT surgery. However, it appears that the effectiveness of TXA in reducing blood transfusion requirements is lacking. Our results do not provide guidance on the optimal timing of TXA or platelet administration, but rather they can help clinicians to re-evaluate the old notions related to bleeding management strategies. Nevertheless, further prospective studies are required.

## 5. Conclusions

This work highlights the critical implications of intraoperative TXA and platelet administration on transfusion requirements of LT patients. LT recipients were still more likely to require more blood product transfusion, despite bleeding management strategies of TXA and platelet administration. Taken collectively, our findings provide incremental evidence and warrant further investigation into the optimal timing of TXA administration to determine the true benefits associated with prophylactic TXA compared to intraoperative TXA administration.

## Figures and Tables

**Table 1:** Baseline characteristics of LT patients categorised by intraoperative treatment of TXA.

	Non-TXA Group (*n* = 76)	TXA Group (*n* = 86)	*p*-Value	Observed Power
Age (years)	58.00 (10.50)	59.00 (9.25)	0.024 *	0.447
Gender				
Male	53 (69.73%)	48 (55.81%)	0.076	-
Female	23 (30.26%)	38 (44.18%)		-
BMI	30.11 (6.98)	30.06 (9.98)	0.163	-
MELD	20.00 (6.75)	21.00 (11.25)	0.204	-
Preoperative Laboratory Variables				
Haemoglobin	112.00 (29.25)	96.00 (30.50)	0.093	-
Platelets	98.50 (68.50)	70.00 (34.25)	0.006 *	0.766
INR	1.40 (0.35)	1.48 (0.57)	0.095	-
Neutrophils	3.89 (3.92)	2.30 (1.73)	<0.001 *	0.770
Lymphocytes	0.91 (0.75)	0.67 (0.76)	0.521	-
Intraoperative Fluid Management (mL)				
Crystalloids	6000.00 (3127.75)	6000.00 (3736.50)	0.207	-
Colloids	975.00 (1037.50)	725.00 (1012.50)	0.491	-

TXA, tranexamic acid; BMI, body mass index; MELD, model for end-stage liver disease; INR, international normalised ratio. Continuous data are presented as the median (interquartile range), and categorical data are presented as number of cases (%). * Statistically significant results from Fisher’s exact test for comparison of categorical variables or Mann–Whitney U-test for comparison of continuous variables.

**Table 2:** Intraoperative transfusion requirements of blood components categorised by intraoperative treatment of TXA.

	Non-TXA Group (*n* = 76)	TXA Group (*n* = 86)	*p*-Value	Observed Power
PRBCs (units)	4.50 (6.00)	8.00 (9.00)	0.001 *	0.588
Platelets	0.00 (12.00)	12.00 (23.00)	<0.001 *	0.969
Cryoprecipitates	0.00 (17.50)	20.00 (29.00)	0.004 *	0.587
FFP	0.50 (4.00)	5.00 (8.00)	<0.001 *	0.912
Fibrinogen concentrate	4.00 (8.00)	4.00 (9.50)	0.063	0.564
Cell savers	0.00 (700.00)	600.00 (1175.00)	0.013 *	0.653

TXA, tranexamic acid; PRBCs, packed red blood cells; FFP, fresh frozen plasma. Data are presented as the median (interquartile range). * Statistically significant from Mann–Whitney U-test results for comparison of continuous variables.

**Table 3:** Postoperative outcomes of LT patients categorised by intraoperative treatment of TXA.

	Non-TXA Group (*n* = 76)	TXA Group (*n* = 86)	*p*-Value	Observed Power
HAT	7 (9.20%)	2 (2.30%)	0.083	-
PVT	3 (3.90%)	2 (2.30%)	0.666	-
Graft loss	6 (7.90%)	14 (16.30%)	0.151	-
Time to extubate (days)	1.00 (0.00)	1.00 (1.00)	0.002 *	0.580
Length of ICU stay (days)	3.00 (2.00)	3.00 (3.25)	0.186	-
90-day mortality	2 (2.60%)	7 (8.10%)	0.171	-

TXA, tranexamic acid; HAT, hepatic artery thrombosis; PVT, portal vein thrombosis. Continuous data are presented as the median (interquartile range), and categorical data are presented as number of cases (%). * Statistically significant results from Mann–Whitney U-test for comparison of continuous variables.

**Table 4:** Demographics and clinical characteristics of LT patients categorised by intraoperative transfusion of platelets.

Variable	No Platelets Group (*n* = 69)	Platelets Group (*n* = 93)	*p*-Value	Observed Power
Age	58.00 (12.00)	58.00 (10.50)	0.963	-
Gender				
Male	43 (62.31%)	58 (62.36%)	1.00	-
Female	26 (37.68%)	35 (37.63%)		-
BMI	28.58 (9.15)	30.94 (7.88)	0.134	-
MELD	20.00 (8.50)	21.00 (9.50)	0.007	0.892
Preoperative Laboratory Variables				
Haemoglobin (g/dL)	112.00 (27.00)	97.00 (34.50)	0.002 *	0.880
Platelets	122.00 (93.00)	69.00 (25.00)	<0.001 *	1.000
INR	1.31(0.26)	1.50 (0.52)	<0.001 *	0.987
Neutrophils	3.84 (4.45)	2.43 (2.28)	0.033 *	0.353
Lymphocytes	1.02 (0.85)	0.70 (0.67)	0.016 *	0.059
Intraoperative TXA				
Yes	26 (37.68%)	60 (64.51%)	<0.001 *	1.000
No	43 (62.31%)	33 (35.48%)		-
Dose	0.00 (1000.00)	1000.00 (1750.00)	<0.001 *	0.852
Intraoperative Fluid Management (mL)				
Crystalloids	5000.00 (2486.00)	6500.00 (3740.00)	0.005 *	0.662
Colloids	1000.00 (1000.00)	750.00 (1150.00)	0.540	-
Transfusion of Blood Components				
PRBCs	2.00 (4.00)	9.00 (8.00)	<0.001 *	1.000
Platelets	-	12.00 (14.00)	-	-
Cryoprecipitates	0.00 (10.00)	20.00 (30.00)	<0.001 *	1.000
FFP	0.00 (3.00)	6.00 (8.00)	<0.001 *	1.000
Fibrinogen concentrate	4.00 (6.00)	8.00 (9.00)	<0.001 *	0.986
Cell saver	0.00 (550.00)	366.00 (1017.00)	0.007 *	0.780

Body mass index; MELD, model for end-stage liver disease; INR, international normalised ratio; TXA, tranexamic acid; PRBCs, packed red blood cells; FFP, fresh frozen plasma. Continuous data are presented as the median (interquartile range), and categorical data are presented as number of cases (%). * Statistically significant from Fisher’s exact test for comparison of categorical variables or Mann–Whitney U-test for comparison of continuous variables.

**Table 5:** Postoperative outcomes of LT patients categorised by intraoperative transfusion of platelets.

	No Platelets Group (*n* = 69)	Platelets Group (*n* = 93)	*p*-Value	Observed Power
HAT	3 (4.34%)	6 (6.45%)	0.733	-
PVT	2 (2.90%)	3 (3.22%)	1.00	-
Time to extubate (days)	1.00 (0.50)	1.00 (1.00)	0.002 *	0.510
Length of ICU stay (days)	2.00 (2.00)	3.00 (3.00)	0.021 *	0.510
Graft loss	8 (11.59%)	12 (12.90%)	0.815	-
90-day mortality	2 (2.90%)	7 (7.53%)	0.302	-

HAT, hepatic artery thrombosis; PVT, portal vein thrombosis. Continuous data are presented as the median (interquartile range), and categorical data are presented as number of cases (%). * Statistically significant results from Mann–Whitney U-test for comparison of continuous variables.

**Table 6:** Demographics and clinical characteristics of LT patients categorised by combined treatment of platelets and TXA.

Variable	No Platelets/TXA Group (*n* = 43)	Platelets/TXA Group (*n* = 60)	*p*-Value	Observed Power
Age	57.00 (12.50)	58.00 (10.00)	0.117	-
Gender				
Male	30 (69.77%)	35 (58.33%)	0.481	-
Female	13 (30.23%)	25 (41.67%)		
BMI	27.51 (7.96)	30.05 (10.26)	0.084	-
MELD	20.00 (9.00)	22.00 (11.50)	0.015 *	0.810
Preoperative Laboratory Variables				
Haemoglobin (g/dL)	118.00 (19.00)	97.00 (30.50)	0.003 *	0.759
Platelets	130.00 (127.00)	69.00 (21.00)	<0.001 *	1.000
INR	1.33(0.33)	1.60 (0.57)	<0.001 *	0.977
Neutrophils	4.00 (3.98)	2.19 (1.64)	<0.001 *	0.663
Lymphocytes	0.97 (0.99)	0.67 (0.56)	0.063	-
TXA dose	-	1000.00 (1000.00)	-	-
Intraoperative Fluid Management (mL)				
Crystalloids	5000.00 (2986.00)	7000.00 (4000.00)	0.015 *	0.450
Colloids	1000.00 (1000.00)	750.00 (1100.00)	0.400	-
Transfusion of Blood Components				
PRBCs	2.00 (3.50)	11.00 (9.00)	<0.001 *	1.000
Platelets	-	18.00 (15.00)	-	-
Cryoprecipitates	0.00 (0.00)	20.00 (30.00)	<0.001 *	0.999
FFP	0.00 (2.50)	8.00 (8.00)	<0.001 *	1.000
Fibrinogen concentrate	2.00 (6.00)	8.00 (10.50)	<0.001 *	0.953
Cell savers	0.00 (550.00)	500.00 (1260.00)	0.003 *	0.850

TXA, tranexamic acid; body mass index; MELD, model for end-stage liver disease; INR, international normalised ratio; PRBCs, packed red blood cells; FFP, fresh frozen plasma. Continuous data are presented as the median (interquartile range), and categorical data are presented as number of cases (%). * Statistically significant findings from Mann–Whitney U-test for comparison of continuous variables.

**Table 7:** Postoperative outcomes of LT patients categorised by combined treatment of platelets and TXA.

	No Platelets/TXA Group (*n* = 43)	Platelets/TXA Group (*n* = 60)	*p*-Value	Observed Power
HAT	2 (4.65%)	1 (1.67%)	0.116	-
PVT	2 (4.65%)	2 (3.33%)	0.689	-
Time to extubate (days)	1.00 (1.00)	1.00 (1.00)	<0.001 *	0.769
Length of ICU stay (days)	3.00 (2.00)	3.00 (4.50)	0.030 *	0.640
Graft loss	4 (9.30%)	10 (16.67%)	0.447	-
90-day mortality	1 (2.32%)	6 (10.00%)	0.149	-

HAT, hepatic artery thrombosis; PVT, portal vein thrombosis. Continuous data are presented as the median (interquartile range), and categorical data are presented as number of cases (%). * Statistically significant results from Mann–Whitney U-test for comparison of continuous variables.

## Data Availability

The data that support the findings of this study are available from KAIMRC, but restrictions apply to the availability of these data, which were used under license for the current study, and so are not publicly available.
